# Correction: Song playbacks demonstrate slower evolution of song discrimination in birds from Amazonia than from temperate North America

**DOI:** 10.1371/journal.pbio.3000568

**Published:** 2019-11-13

**Authors:** The PLOS Biology Staff

## Notice of republication

This article was republished on 30^th^ October 2019, to replace Table 1 as the bottom portion of the table, and the caption, were inadvertently omitted in the PDF version of the article. The publisher apologizes for this error.

Please download this article again to view the correct version. The originally published, uncorrected article is provided here for reference.

## Supporting information

S1 File Originally published, uncorrected article. (PDF)
