# Supplementary figures and images for: Song playbacks demonstrate slower evolution of song discrimination in birds from Amazonia than from temperate North America
Source: PLoS Biol. 2019 Oct 22;17(10):e3000478. doi: 10.1371/journal.pbio.3000478 (PMC6804960; doi:10.1371/journal.pbio.3000478)

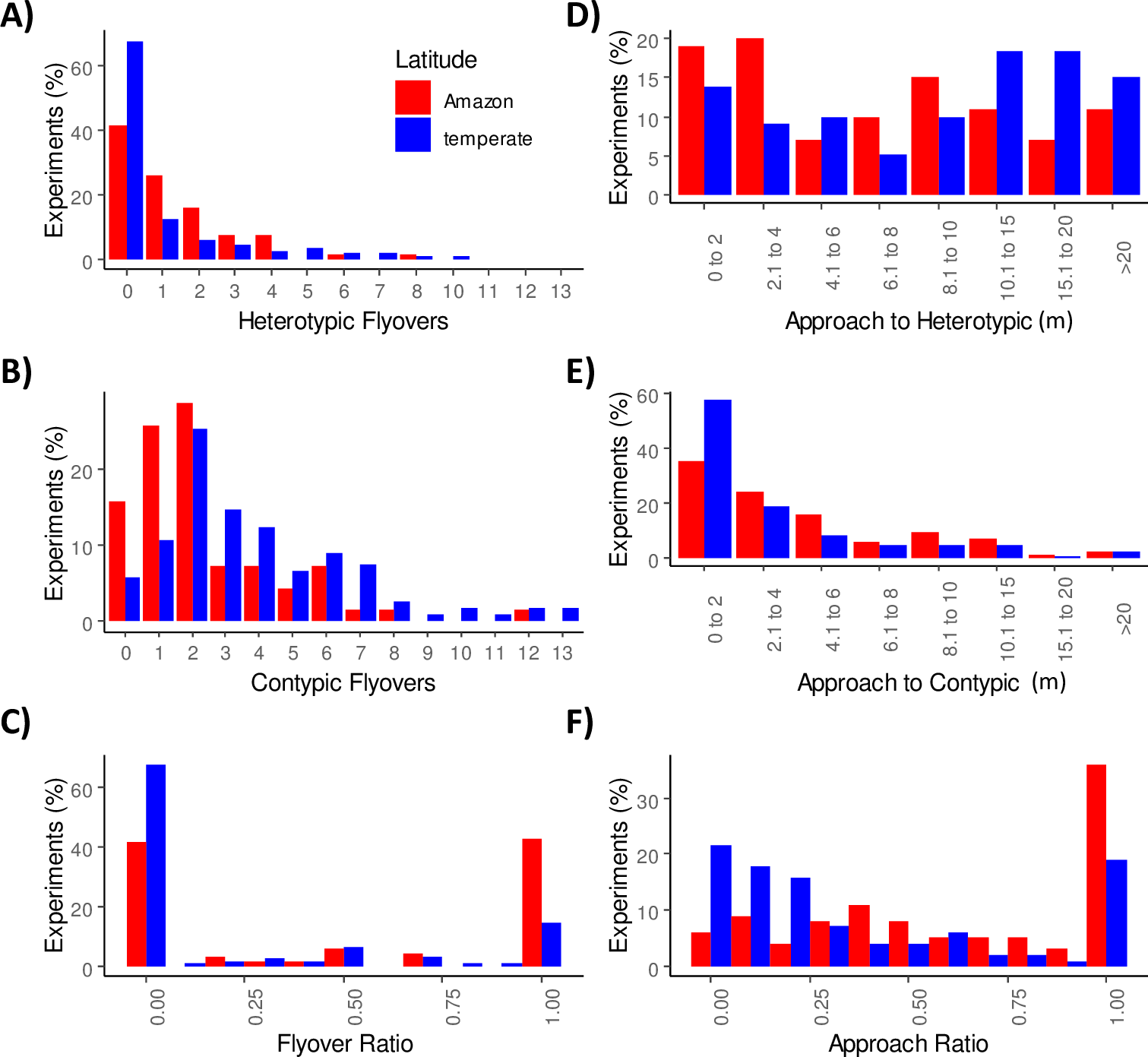

Supplement: S1 Fig — Left: the number of flyovers and flybys across the speaker during playback of (A) heterotypic and (B) contypic songs and (C) the ratio between A and B used to quantify relative level of heterotypic discrimination. Ratios greater than 1 are set to 1 indicating a lack of discrimination, while values of 0 indicate response only to conspecific playback. Right: the closest approach to the speaker during playback of (D) heterotypic and (E) contypic song and (F) the ratio of conspecific to heterospecific approach as an indicator of heterospecific discrimination used to quantify relative level of heterotypic discrimination. Ratios of 0 indicate approach only to conspecific. Ratios of 1 indicate equal response to conspecific and heterospecific. Data used to make this figure are in S1 Data. (TIF) [file pbio.3000478.s001.tif]

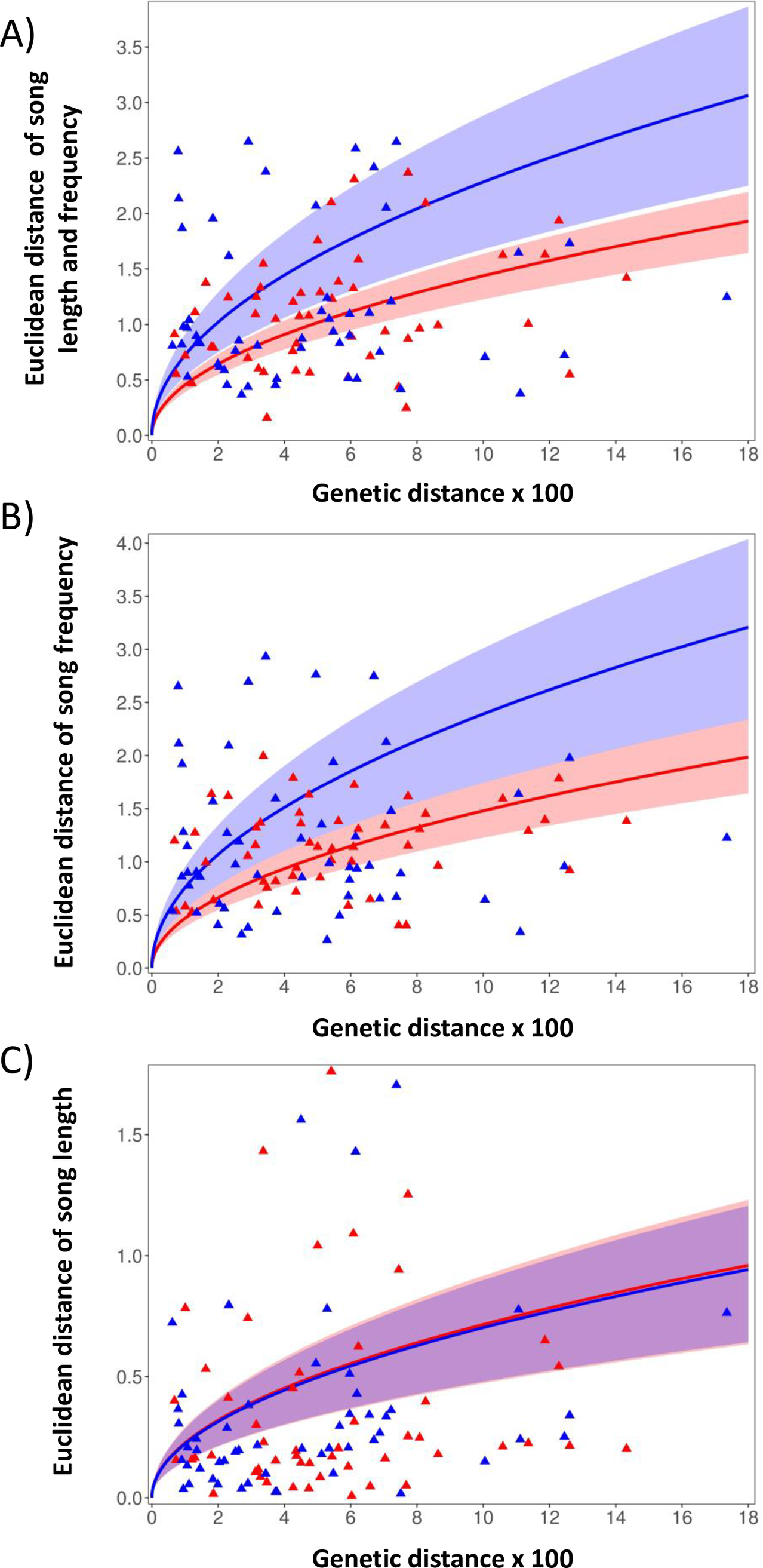

Supplement: S2 Fig — Song evolves faster at higher latitudes for (A) overall song divergence as measured by Euclidean distances of PC1 to PC3 for 12 measures of song timing and frequency and (B) song frequency as measured by Euclidean distances for PC1 to PC3 for 10 measures of frequency but not (C) Euclidean distances of log-transformed song length. Lines show the mean evolutionary rate (δ2) under a Brownian motion model obtained from 1,000 datasets that randomly sampled with replacement one sister pair per species complex. Shading show 95% confidence bands obtained from 1,000 bootstrap replicates. Data used to make this figure are in S1 Data. PC, principal component. (TIF) [file pbio.3000478.s002.tif]

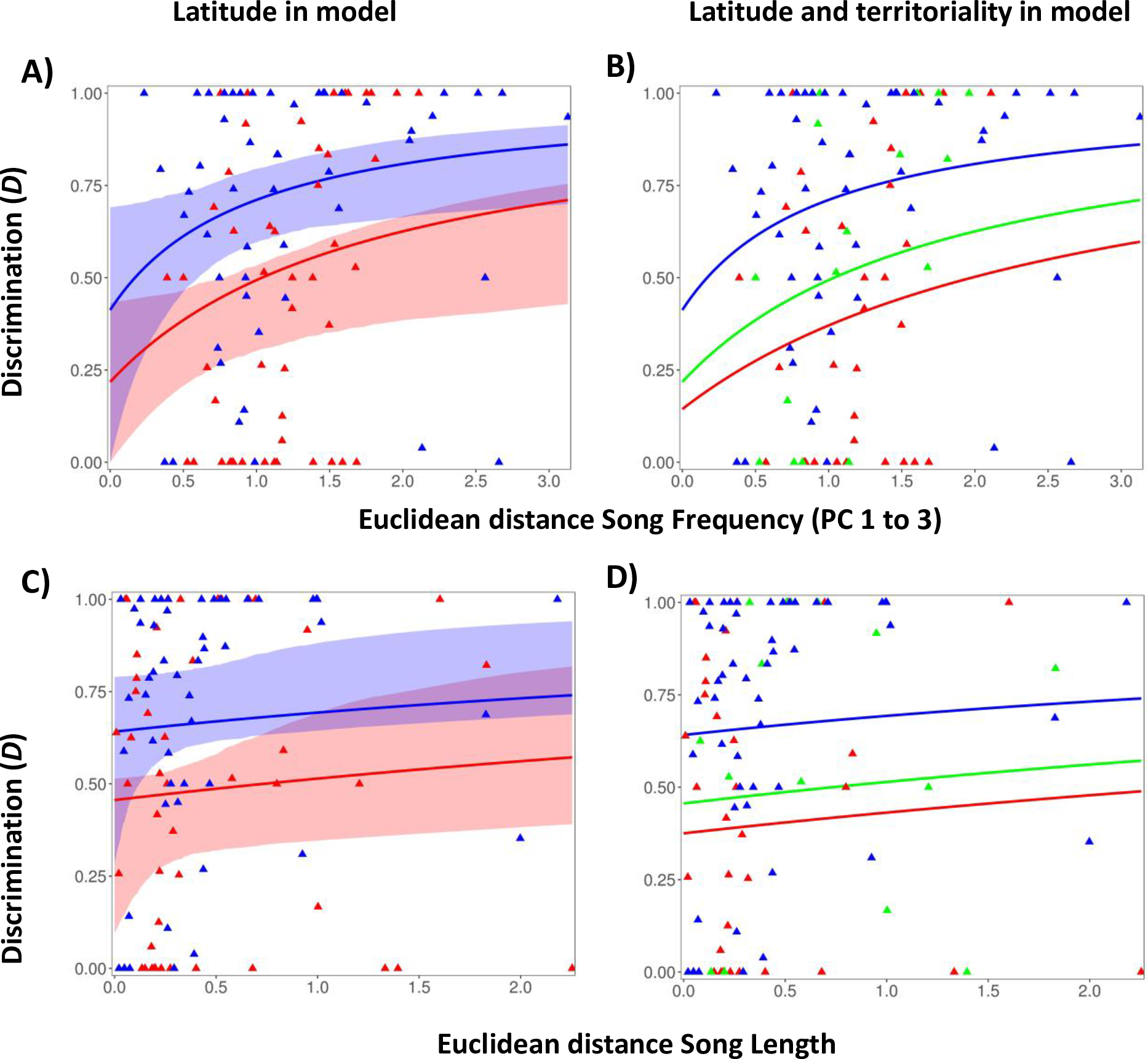

Supplement: S3 Fig — Song divergence is measured using a Euclidean distance of PC1 to PC3 obtained from 10 measures of frequency and its autocorrelation (A), and song length is measured using the Euclidean distance of log-transformed song lengths (B). Lines and shading as in Fig 1. Though confidence bands overlap across bootstraps, discrimination was greater in the temperate than Amazonia in 100% of bootstrap replicates for (A), 97.1% for (B), 100% for (C), and 95% for (D). Data used to make this figure are in S1 Data. PC, principal component. (TIF) [file pbio.3000478.s003.tif]

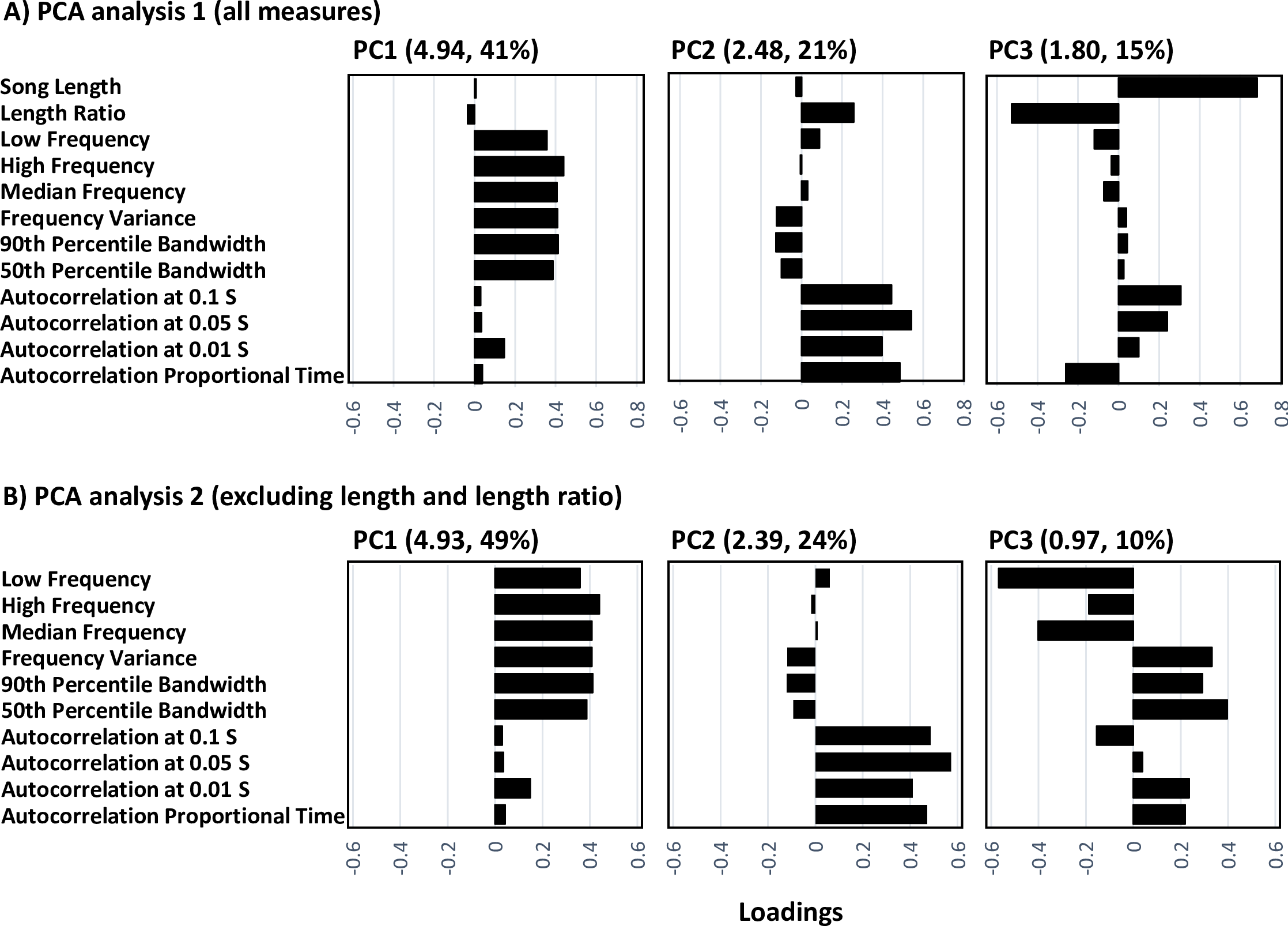

Supplement: S4 Fig — Loadings for PCA of (A) all 12 measures of song frequency and length and (B) 10 measures of frequency and its autocorrelation. Eigenvalues and percent variance explained by each PC are indicated above each panel. PC, principal component; PCA, principal component analysis. (TIF) [file pbio.3000478.s004.tif]

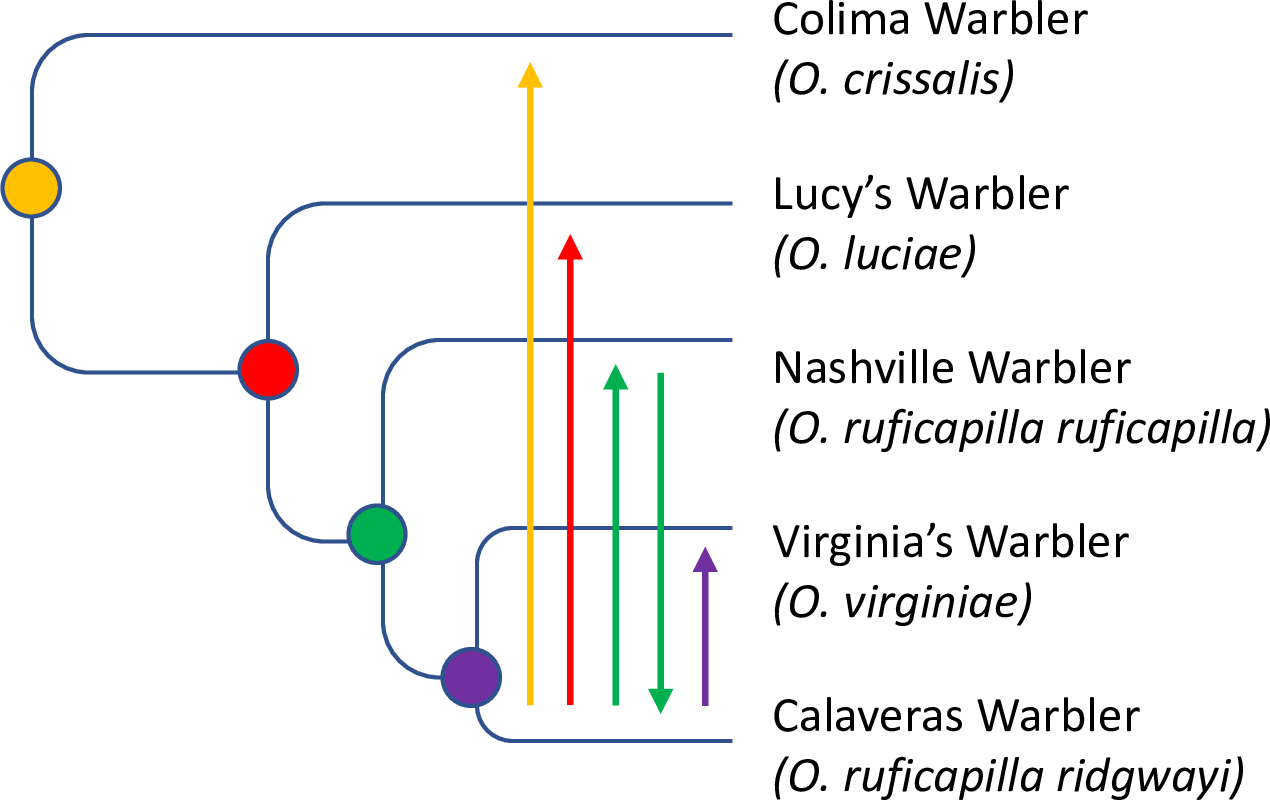

Supplement: S5 Fig — Arrows start at the taxon on which playback was performed and point to the taxon whose song was tested. Arrow colors correspond to their corresponding colored nodes on the phylogeny. For the Nashville Warbler and Calaveras Warbler species pair, playbacks were performed on both species. In all such cases, the average response was used as the metric of discrimination for the species pair. Nested species pairs are not statistically independent. Thus, for each analysis in this paper, 1,000 datasets were generated, each with a single taxon pair drawn at random from each species complex. Statistics were then performed on these datasets. For bootstrap analysis, a single bootstrapped dataset was generated from each randomized dataset. (TIF) [file pbio.3000478.s005.tif]
