# Supplementary material for: Song playbacks demonstrate slower evolution of song discrimination in birds from Amazonia than from temperate North America
Source: PLoS Biol. 2019 Oct 22;17(10):e3000478. doi: 10.1371/journal.pbio.3000478 (PMC6804960; doi:10.1371/journal.pbio.3000478)
Supplement: S1 Table — (DOCX) [file pbio.3000478.s006.docx]

| **S1 Table** |  |  |
| --- | --- | --- |
| Social system classification of the pairs in the study [modified from [1], as indicated in the supplemental dataset]. In all but one case *(Icterus)* both members of the pair had the same social system; in the one case the control (Bullock’s Oriole) is counted. | | |
| Territoriality | Amazon | North America |
| Not territorial | 7 | 1 |
| Seasonal | 9 | 54 |
| Year round | 35 | 3 |
| Total | 52 | 58 |
|  |  |  |

**References**

Tobias J, Sheard C, Seddon N, Meade A, Cotton A, Nakagawa S. Territoriality, social bonds, and the evolution of communal signaling in birds. Front Ecol Evol. 2016;4:
