# Supplementary material for: Song playbacks demonstrate slower evolution of song discrimination in birds from Amazonia than from temperate North America
Source: PLoS Biol. 2019 Oct 22;17(10):e3000478. doi: 10.1371/journal.pbio.3000478 (PMC6804960; doi:10.1371/journal.pbio.3000478)
Supplement: S2 Table — (DOCX) [file pbio.3000478.s007.docx]

**S2 Table.** Support for models of song discrimination evolution as a function of genetic distance using the Michaelis-Menten modelling framework for sister pairs with two or more playback experiments. All values and ranges calculated as for Table 1.

| **Model** | **N** | **Akaike Weight** | **β North America** | **β Amazon** |
| --- | --- | --- | --- | --- |
| *a) all sister pairs (n=66)* |  |  |  |  |
| 1. Null | 1 | 0.000 (0.000) | 2.03 (1.16-3.51) | Same |
| 1. temperate / Amazon | 2 | 0.042 (0.011) | 1.06 (0.53-1.82) | 6.00 (2.49-12.66) |
| 1. learned / innate | 2 | 0.013 (0.006) | 0.99 (0.51-1.54) | 4.73 (2.10-8.02) |
| 1. temperate / Amazon for learned / innate | 4 | 0.016 (0.006) | 0.94 (0.49-1.41) / 2.53 (0.00-7.83) | 10.84 (8.22-14.33) / 5.60 (2.21-10.45) |
| 1. presence / absence of year-round territoriality | 2 | 0.274 (0.033) | 7.13 (3.17-12.08) / 1.06 (0.56-1.57) | Same |
| 1. temperate / presence year-round territoriality Amazon / absence year-round territoriality Amazon | 3 | 0.654 (0.055) | 1.06 (0.53-1.59) | 8.99 (3.98-16.67) / 1.29 (0.21-3.64) |
