# Supplementary material for: Song playbacks demonstrate slower evolution of song discrimination in birds from Amazonia than from temperate North America
Source: PLoS Biol. 2019 Oct 22;17(10):e3000478. doi: 10.1371/journal.pbio.3000478 (PMC6804960; doi:10.1371/journal.pbio.3000478)
Supplement: S3 Table — (DOCX) [file pbio.3000478.s008.docx]

**S3 Table.** Support for models of song evolution as a function of genetic distance using Brownian motion models for: a) song length (using Euclidean distance of log transformed song length), b) song frequency (using Euclidean distance of PC1 to PC3 derived from 10 measurements of song frequency and its autocorrelation), c) combined (using Euclidean distance of PC1 to PC3 of song length and frequency measurements). All values and ranges calculated as for Table 1. σ^2^ is the evolutionary rate.

| **Model** | **N** | | **Akaike Weight** | **σ^2^ North America** | **σ^2^ Amazon** |
| --- | --- | --- | --- | --- | --- |
| *a) Song Length (n=104)* | |  |  |  |  |
| 1. Null | 1 | | 0.470 (0.035) | 0.039 (0.023-0.057) | same |
| 1. temperate / Amazon | 2 | | 0.180 (0.019) | 0.038 (0.017-0.064) | 0.040 (0.016-0.066) |
| 1. learned / innate | 2 | | 0.283 (0.046) | 0.044 (0.022-0.071) / 0.033 (0.013-0.058) | same |
| 1. temperate / Amazon for learned / innate | 4 | | 0.068 (0.009) | 0.039 (0.015-0.069) / 0.033 (0.006-0.078) | 0. 069 (0.008-0.153) / 0. 033 (0.010-0.061) |
|  |  | |  |  |  |
| *b) Song Frequency (n=104)* |  | |  |  |  |
| 1. null | 1 | | 0.032 (0.024) | 0.316 (0.194-0.478) | same |
| 1. temperate / Amazon | 2 | | 0.787 (0.049) | 0.444 (0.223-0.733) | 0.172 (0.118-0.246) |
| 1. learned / innate | 2 | | 0.051 (0.021) | 0.392 (0.185-0.0.672) / 0.222 (0.147-0.312) | same |
| 1. temperate / Amazon for learned / innate | 4 | | 0.130 (0.007) | 0.447 (0.204-0.770) / 0.425 (0.084-0.817) | 0.130 (0.080-0.187) / 0.182 (0.118-0.278) |
| *c) Song Combined (n=104)* |  | |  |  |  |
| 1. null | 1 | | 0.034 (0.018) | 0.294 (0.191-0.439) | same |
| 1. temperate / Amazon | 2 | | 0.737 (0.036) | 0.411 (0.222-0.677) | 0.163 (0.118-0.216) |
| 1. learned / innate | 2 | | 0.097 (0.019) | 0.375 (0.188-0.639) / 0.193 (0.140-0.257) | same |
| 1. temperate / Amazon for learned / innate | 4 | | 0.132 (0.006) | 0.427 (0.209-0.742) / 0.304 (0.131-0.474) | 0.127 (0.067-0.189) / 0.172 (0.120-0.237) |
