# Supplementary material for: Song playbacks demonstrate slower evolution of song discrimination in birds from Amazonia than from temperate North America
Source: PLoS Biol. 2019 Oct 22;17(10):e3000478. doi: 10.1371/journal.pbio.3000478 (PMC6804960; doi:10.1371/journal.pbio.3000478)
Supplement: S4 Table — (DOCX) [file pbio.3000478.s009.docx]

**S4 Table.** Support for models of song discrimination evolution as a function of song divergence (see equation 4 in the text) measured as: a) song length (using Euclidean distance of log transformed song length), b) song frequency (using Euclidean distance of PC1 to PC3 derived from 10 measurements of song frequency and its autocorrelation), c) combined (using Euclidean distance of PC1 to PC3 of song length and frequency measurements). Mean Akaike Weights (standard deviation) and β are shown from 1000 randomizations whereby one taxon pair was chosen randomly from nested subsets within each species complex (see Supplementary Fig. 2).

| **Model** | **N** | | **Akaike Weight** | | | **β North America** | **β Amazon** | **z** |
| --- | --- | --- | --- | --- | --- | --- | --- | --- |
| *a) Song Length (n=104)* | | | |  |  |  |  |  |
| 1. Null | 1 | | 0.008 (0.004) | | | -0.530 | same | -0.53 |
| 1. temperate / Amazon | 2 | | 0.557 (0.028) | | | -1.688 | 1.635 | -3.23 |
| 1. learned / innate | 2 | | 0.011 (0.0034) | | | -1.297 / 0.525 | same | -3.60 |
| 1. temperate / Amazon for learned / innate | 4 | | 0.109 (0.014) | | | -1.464 / -2.050 | 3.023 / 1.236 | -2.97 |
| 1. presence / absence of year-round territoriality | 2 | | 0.062 (0.020) | | | -1.803 / -2.128 | same | -4.40 |
| 1. temperate / absence year-round territoriality Amazon / presence year-round territoriality Amazon | 3 | | 0.253 (0.018) | | | -2.035 | 0.709 / 2.549 | -3.84 |
|  |  | |  | | |  |  |  |
| *b) Song Frequency (n=104)* | |  |  | | |  |  |  |
| 1. null | 1 | | 0.026 (0.058) | | | -0.038 | same | -0.04 |
| 1. temperate / Amazon | 2 | | 0.329 (0.183) | | | -0.946 | 2.059 | -2.29 |
| 1. learned / innate | 2 | | 0.029 (0.057) | | | -0.492 / 1.155 | same | -2.21 |
| 1. temperate / Amazon for learned / innate | 4 | | 0.145 (0.151) | | | -1.051 / -1.451 | 4.130 / 2.008 | -2.60 |
| 1. presence / absence of year-round territoriality |  | | 0.139 (0.170) | | | -0.462 / -2.111 | same | -1.95 |
| 1. temperate / absence year-round territoriality Amazon / presence year-round territoriality Amazon |  | | 0.332 (0.190) | | | -0.786 | 1.099 / 2.722 | -1.99 |
| *c) Song Combined (n=104)* | |  |  | | |  |  |  |
| 1. null | 1 | | 0.027 (0.058) | | | -0.021 | same | -0.02 |
| 1. temperate / Amazon | 2 | | 0.326 (0.185) | | | -0.968 | 2.100 | -2.37 |
| 1. learned / innate | 2 | | 0.028 (0.050) | | | -0.486 / 1.150 | same | -2.29 |
| 1. temperate / Amazon for learned / innate | 4 | | 0.147 (0.159) | | | 3.191 / 2.801 | 6.547 / 5.633 | -2.64 |
| 1. presence / absence of year-round territoriality | 2 | | 0.147 (0.172) | | | -0.463 / 2.180 | same | -1.99 |
| 1. temperate / absence year-round territoriality Amazon / presence year-round territoriality Amazon | 3 | | 0.324 (0.183) | | | -0.783 | 1.029 / 2.783 | -2.03 |
