# Supplementary material for: Correction: Song playbacks demonstrate slower evolution of song discrimination in birds from Amazonia than from temperate North America
Source: PLoS Biol. 2019 Nov 13;17(11):e3000568. doi: 10.1371/journal.pbio.3000568 (PMC6853300; doi:10.1371/journal.pbio.3000568)
Supplement: S1 File — (PDF) [file pbio.3000568.s001.pdf]

PLOS BIOLOGY

SHORT REPORTS

Song playbacks demonstrate slower evolution of song discrimination in birds from Amazonia than from temperate North America

Jason T. Weir[1,2,3]*, **Trevor D. Price**[1]

1 Department of Ecology and Evolution, University of Chicago, Chicago, Illinois, United States of America, 2 Department of Ecology and Evolutionary Biology and Department of Biological Sciences, University of Toronto, Toronto, Canada, 3 Department of Natural History, Royal Ontario Museum, Toronto, Canada

* jason.weir@utoronto.ca

Check for updates

OPEN ACCESS

Citation: Weir JT, Price TD (2019) Song playbacks demonstrate slower evolution of song discrimination in birds from Amazonia than from temperate North America. PLoS Biol 17(10): e3000478. https://doi.org/10.1371/journal.pbio.3000478

Academic Editor: Hélène Morlon, Ecole Normale Supérieure, FRANCE

Received: April 18, 2019

Accepted: September 17, 2019

Published: October 22, 2019

Copyright: © 2019 Weir, Price. This is an open access article distributed under the terms of the Creative Commons Attribution License, which permits unrestricted use, distribution, and reproduction in any medium, provided the original author and source are credited.

Data Availability Statement: All relevant data are within the paper and its Supporting Information files, which we have uploaded.

Funding: Funding supplied by a National Geographic Waitt Grant (https://www.waittfoundation.org/national-geographic-society-grants), Natural Sciences and Engineering Research Council of Canada (NSERC, http://www.nserc-crsng.gc.ca) Discovery Grants 402013-2011 and RGPIN-2016-06538, and NSERC Discovery Accelerator Grant 492890 to JTW and a National

Abstract

Genetic data indicate differences in speciation rate across latitudes, but underlying causes have been difficult to assess because a critical phase of the speciation process is initiated in allopatry, in which, by definition, individuals from different taxa do not interact. We conducted song playback experiments between 109 related pairs of mostly allopatric bird species or subspecies in Amazonia and North America to compare the rate of evolution of male discrimination of songs. Relative to local controls, the number of flyovers and approach to the speaker were higher in Amazonia. We estimate that responses to songs of relatives are being lost about 6 times more slowly in Amazonia than in North America. The slow loss of response holds even after accounting for differences in song frequency and song length. Amazonian species with year-round territories are losing aggressive responses especially slowly. We suggest the presence of many species and extensive interspecific territoriality favors recognition of songs sung by sympatric heterospecifics, which results in a broader window of recognition and hence an ongoing response to novel similar songs. These aggressive responses should slow the establishment of sympatry between recently diverged forms. If male responses to novel allopatric taxa reflect female responses, then premating reproductive isolation is also evolving more slowly in Amazonia. The findings are consistent with previously demonstrated slower recent rates of expansion of sister taxa into sympatry, slower rates of evolution of traits important for premating isolation, and slower rates of speciation in general in Amazonia than in temperate North America.

Introduction

In the Americas, recent speciation rates in birds and mammals, as assessed from genetic data, have been slower at lower latitudes [1]. Avian sister pairs of the same age in the New World are less divergent in the tropics than the temperate region in body mass [2], song frequency [2,3], song complexity [4], and color [5]. All of these traits have been shown to be the basis for female choice of conspecifics in experimental studies and thereby contribute to premating

Science Foundation Grant (https://www.nsf.gov/) 0640139 to TP. The funders had no role in study design, data collection and analysis, decision to publish, or preparation of the manuscript.

Competing interests: The authors have declared that no competing interests exist

Abbreviations: CC, Creative Commons; COI, cytochrome oxidase I; Cyt b, cytochrome b; ND2, NADH dehydrogenase 2; PC, principal component; PCA, principal component analysis.

reproductive isolation [6]. Reproductive isolation generally starts to develop during a phase when populations are geographically separated from each other [6,7]. If sufficient reproductive isolation arises, populations will not merge when they come into contact (i.e., they are species). The development of reproductive isolation during the allopatric phase is difficult to study because individuals from the different taxa do not encounter each other in nature. Hence, this critical part of the speciation process remains largely unexplored. In birds, a tractable approach to the problem has been through the playback of song to males. Males holding territories respond aggressively to playback of songs from local males [8]. A strong response of the local male to a song from a related allopatric taxon is taken to indicate that the song is recognized as belonging to a conspecific. Accordingly, it is assumed that little reproductive isolation has developed, and it is likely that the two forms would interbreed on first contact [9–12]. By contrast, a weak response may be used to conclude the two forms are different species. These inferences are supported by the demonstration that named subspecies—assumed to be further along in the speciation process than populations that have not been given subspecies status—often exhibit reduced response to playback [8] and that song responses are negatively correlated with divergence in song traits [12]. In this paper, we use song playbacks to study rates of evolution of male responses across latitudes by conducting identical experiments in tropical Amazonia and in temperate North America.

In the Americas, in addition to speciation rate, sympatry between avian sister species has been achieved more slowly at lower latitudes than at higher latitudes [13,14]. While aggressive responses to playback are regularly taken to indicate the progress of premating reproductive isolation, male aggression is also recognized as directly contributing to the setting of range limits [15]. In birds, aggression between males can retard expansion into sympatry of divergent taxa by preventing foreign males from establishing territories [16,17]. Establishment of extensive sympatry itself contributes to the completion of premating reproductive isolation through processes such as reinforcement (selection against cross-taxon matings driven by low hybrid fitness [18,19]) and other kinds of reproductive interference [20,21]. Hence, male responses to related allopatric taxa should indicate not only the rate of development of reproductive isolation in allopatry but also how reproductive isolation may be further affected by slowing the rate at which different forms come into sympatry.

Limits to the establishment of sympatry are easier to infer from playback experiments than the development of reproductive isolation in allopatry. Differences in male responses to playback between latitudes lead us to directly infer differences in limits on range expansions. On the other hand, inferences on female propensity to hybridize assume that male aggressive responses are correlated with a female's tendency to mate with males of the allopatric taxon. The tropics differ from the temperate in ways that may lead to different strengths of this correlation at different latitudes. First, a lack of strong seasonality in tropical regions means that many species hold year-round territories and interact over long time periods with both conspecifics and sympatric heterospecifics [22]. Second, the Amazon basin contains the greatest bird species richness on earth, with estimates of 160 bird species breeding at one location, more than 4× that of temperate North America [23]. Amazonian species may have similar songs [24] promoted by interspecific interactions [25,26] and presumably also the large number of species occupying an especially restricted acoustic space because of noise from other taxa such as insects [3,27]. Together, the presence of many species and year-round territoriality may select for males to aggressively respond to a wide variety of songs but for females to discriminate between very similar songs [28]. This could decouple male and female responses to allopatric relatives in the tropics to a greater extent than in the temperate. However, evidence from hybrid zones, as summarized in the discussion, indicates that premating isolation is likely to correlate positively with male aggressive responses, even in comparisons across latitudes.

We compared the response of males to playback of the territorial song of a closely related, generally allopatric taxon to that of its own taxon's song [8,12]. We performed 245 playback experiments between 109 closely related pairs of bird species or subspecies across temperate North America (58 pairs) and the tropical lowlands of Amazonia (51 pairs). By conducting these experiments across many replicate taxon pairs, each differing in the length of time they have been separated from a common ancestor, we determine that tropical Amazonian species are retaining aggressive responses over a much longer time period than temperate species; i.e., in Amazonia, males respond strongly to songs from quite distantly related allopatric taxa. We asked whether this effect was associated with the prevalence of year-round territoriality in the tropics. While we found that the holding of year-round territories was especially important in slowing the loss of aggressive responses, even after controlling for this, heterospecific responses are retained for longer in Amazonia than at high latitudes. We argue that high aggressive responses in Amazonia should limit range expansions and also imply a slower evolution of premating reproductive isolation. These effects should contribute to the currently slower accumulation of closely related species in sympatry [13,14], and lower rates of speciation towards the equator [1].

Results

Responses to song

We measured two different responses: the number of flights past the speaker and the nearest approach distance while perched or on the ground. Raw data are summarized in S1 Fig. First, individuals responded to heterotypic song by making one or more flights near the speaker almost twice as often in Amazonia as at high latitudes. In terms of the total number of flights near the speaker, the ratio of heterospecific to conspecific responses was about one-half in Amazonia and one-quarter in temperate birds. Second, birds approached the speaker during heterotypic playback more closely in Amazonia. Among individuals that approached during heterotypic playback, in Amazonia, 39% of all individuals came within 4 m and 71% within 10 m during a trial; corresponding figures for the temperate are 23% and 48%, respectively. When heterotypic to contypic responses are compared in average approach distance, Amazonian birds came about two-thirds closer in response to heterotypic playback when compared to contypic playback, but only one-third closer at higher latitudes. We interpret both the results on flights and approach distance to indicate that Amazonian species respond relatively strongly to related forms when compared to temperate species.

For each taxon pair, we computed a single measure of response, *D*, that contrasted both flights and approach distance to the related taxon's playback versus own taxon playback (see Methods). A value of one implies a complete lack of response, i.e., that heterotypic song is ignored, and a value of zero that response to heterotypic song is the same as response to contypic song. Following Freeman and colleagues [29], we quantified the accumulation of *D* with genetic distance using a modeling framework based on the asymptotic Michaelis–Menten curve. We compared the likelihoods of a model in which a single accumulation curve was fitted to all taxon pairs to a model in which separate curves were fitted to Amazonian versus temperate North American taxon pairs (Table 1). The latter model greatly outperformed the former, demonstrating a slower rate of loss of response to heterotypic song in Amazonia (Fig 1A). We also fitted models with separate curves for taxon pairs with innate versus learned song or that defended year-round territories versus those that do not (i.e., were seasonally territorial or nonterritorial) and found poor fits to these models when latitude is not included (Table 1). We fitted the song inheritance model because song learning has previously been reported to result in different rates of accumulation of song responses through time [29], and our

Table 1. Support for latitude versus presence or absence of song learning and year-round territoriality as drivers of song response evolution. Song response is modeled as a function of genetic distance using the Michaelis–Menten framework. The "allopatric" dataset includes allopatric and parapatric taxon pairs but excludes 20 sister pairs that occur in sympatry.

Model	*N*	Akaike Weight	β North America	β Amazon
a) All sister pairs (n = 109)				
1) Null	1	0.000 (0.000)	2.67 (1.60–4.34)	Same
2) Temperate/Amazon	2	0.296 (0.058)	1.14 (0.62–1.79)	6.60 (3.50–11.82)
3) Learned/innate	2	0.000 (0.000)	1.74 (0.90–2.87)/3.98 (1.98–6.21)	Same
4) Temperate/Amazon for learned/innate	4	0.080 (0.023)	1.33 (0.70–2.28)/0.64 (0.07–1.54)	10.60 (1.32–20.00)/6.22 (3.18–9.84)
5) Presence/absence of year-round territoriality	2	0.049 (0.023)	7.33 (3.88–11.36)/1.51 (0.88–2.27)	Same
6) Temperate/absence year-round territoriality Amazon/presence year-round territoriality Amazon	3	0.575 (0.063)	1.14 (0.62–1.79)	9.14 (4.57–15.34)/3.38 (1.27–7.24)
b) Allopatric sister pairs only (n = 89)				
1) Null	1	0.002 (0.002)	3.54 (1.92–6.04)	Same
2) Temperate/Amazon	2	0.280 (0.057)	1.37 (0.65–3.05)	7.24 (3.61–14.34)
3) Learned/innate	2	0.001 (0.001)	2.60 (1.13–4.90)/4.40 (1.95–7.29)	Same
4) Temperate/Amazon for learned/innate	4	0.130 (0.045)	1.91 (0.77–3.78)/0.54 (0.02–1.43)	10.60 (1.54–20.00)/6.84 (3.32–11.60)
5) Presence/absence of year-round territoriality	2	0.209 (0.067)	8.76 (4.47–14.45)/1.84 (0.92–3.04)	Same

(*Continued*)

Amazonian dataset possessed a smaller proportion of taxon pairs with learned song than the North American dataset.

Finally, we fitted models that incorporated song inheritance and latitude together or that allowed separate rates for territorial system in the Amazon and a single rate applied to all taxon pairs in temperate North America (which had only three pairs with year-round territoriality; S1 Table). The three models that included the effect of latitude together accounted for greater than 95% of the Akaike weight, while the three models that did not include latitude had a combined weight of only 5% (Table 1). Rates of loss of response to heterotypic playback are lower in Amazonia than in temperate North America across all three models that include latitude (Fig 1A, Table 1). Of these three models, the model that allowed separate rates for the presence or absence of year-round territoriality in Amazonia received the best fit. Within Amazonia, year-round territoriality is particularly strongly associated with a slow rate of loss of response compared to the alternative nonterritoriality/seasonal territoriality systems (Table 1).

The constant of the Michaelis–Menten curve, β, measures the time point at which response is half of its maximum potential value of 1.0. Smaller values of β thus indicate faster rates at which species response is lost. Under the best-fit model (model 6 in Table 1), β was eight times lower at high latitudes (1.1) compared to Amazonian species that possessed year-round

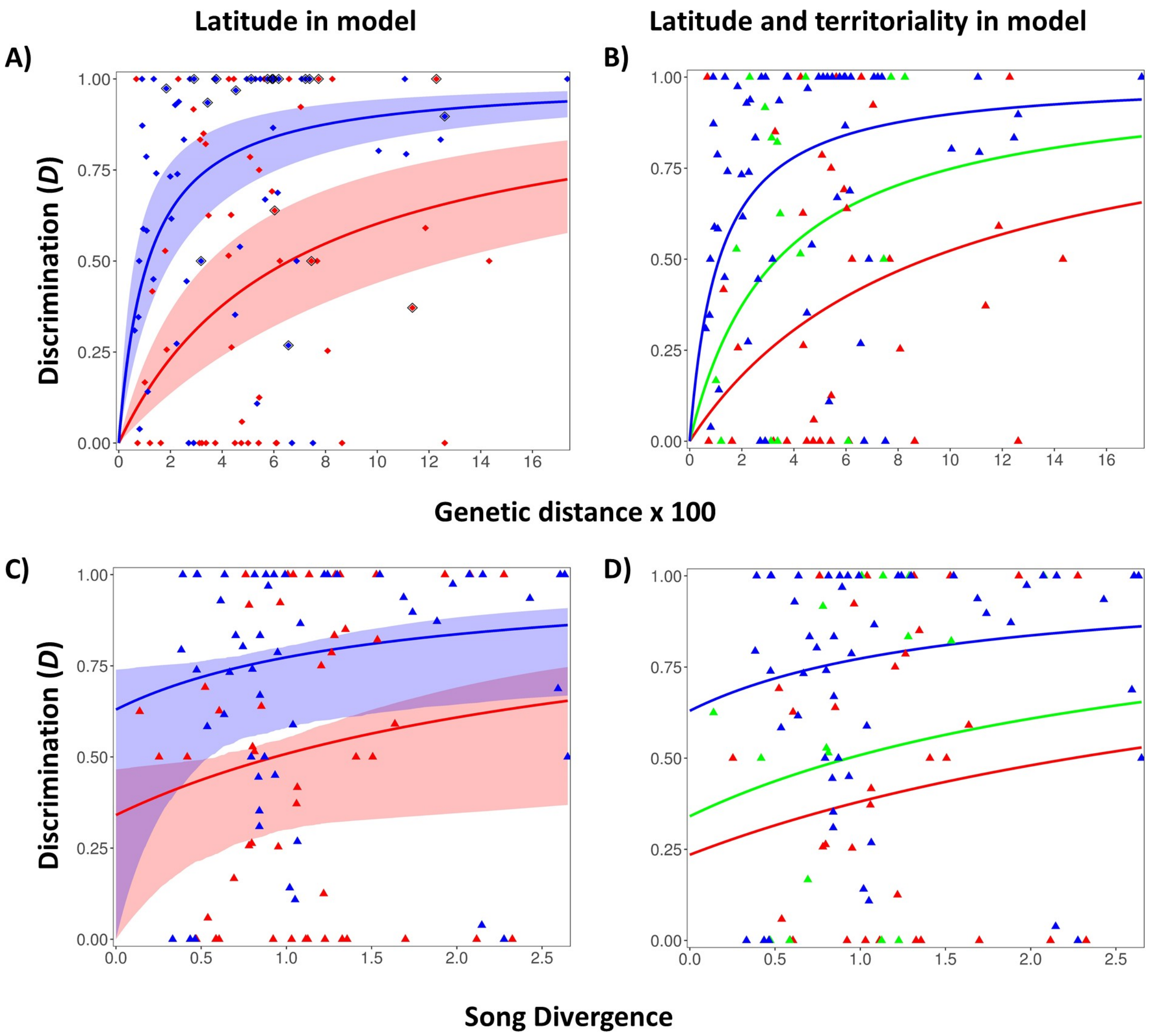

Fig 1. Comparison of song discrimination in Amazonia (red or red and green) versus high latitudes of North America (blue). (A) Song discrimination as a function of genetic distance with separate Michaelis–Menten curves applied to Amazonia and temperate regions. Expectations (solid lines) and 95% confidence bands (shading) obtained from 1,000 bootstrap replicates in which only a single taxon pair was sampled from within each species complex (see S5 Fig). Sympatric taxa pairs that were excluded from analyses using only non-sympatric taxa (see Table 1) are outlined in black. (B) is the same as in A, but with curves for Amazonian species in which year-round territoriality is present (red) or absent (green). (C) and (D) are the same as in A and B, except that song discrimination is a function of a composite of song frequency and length divergence (using Euclidean distance of PC1 to PC3 for our PCA of all song measures) using a Michaelis–Menten curve with intercept free to vary (Eq 4). Though confidence bands overlap across bootstraps in some of these analyses, discrimination was greater in the temperate than Amazonia in 100% of bootstrap replicates for (A), 97.6% for (B), 99.9% for (C), and 95.1% for (D). Data used to make this figure are in S1 Data. PC, principal component; PCA, principal component analysis.

https://doi.org/10.1371/journal.pbio.3000478.g001

territoriality (9.1) and three times lower than Amazonian species that lacked year-round territoriality (3.4). We obtained the same latitudinal patterns and similar rates when excluding the 20 sympatric taxon pairs (Table 1B), indicating that these rate differences hold strongly during the allopatric phase of the speciation process.

Point estimates of song responses based on only a single playback experiment are unbiased but are less precise than when using the average response obtained from multiple experiments, and this lack of precision should contribute to the scatter observed around the best-fit curves

in Fig 1A. Scatter is greatly reduced when restricting our analysis to only taxon pairs with two or more playback experiments averaged (Fig 2). Resulting rate differences between Amazonia and high latitudes remain almost identical, with high latitude rates of response loss being significantly and substantially faster than in Amazonia (β = 1.1 high latitudes; β = 6.0 Amazon; S2 Table).

Song traits

Weaker responses in temperate species may be a consequence of greater divergence in song traits, which are known to differ more among temperate sister species than tropical ones possessing similar degrees of genetic divergence [3,4]. In the present dataset, song frequency differences have accumulated with time more quickly in temperate species than in those from Amazonia, but the rate of accumulation of differences in song length does not differ significantly (S2 Fig, S3 Table).

For a given degree of divergence in song length, in frequency, and in a combined metric of both frequency and length, song responses are lower in temperate than in Amazonian species (Fig 1B, S3 Fig, S4 Table). These results suggest that responses are not being lost more quickly in temperate species simply in response to faster divergence of song there. Rather, response for a given amount of song divergence may itself be being lost more quickly at high latitudes. We estimate response with respect to song divergence is happening particularly slowly in Amazonian species with year-round territories when compared to those without year-round territories (S4 Table).

Discussion

Male responses to a related allopatric taxon's song are being lost more slowly in Amazonia than in temperate North America. Applying the standard 2% mitochondrial molecular clock for birds [30], the halfway point of response loss requires 3.3 million years on average in Amazonia but just 550,000 years at high latitudes in North America. High rates of loss of response in temperate North America have resulted in a number of recently diverged taxa with very weak responses to allopatric forms (Figs 1A and 2). For example, the eastern and western forms of the Common Yellowthroat (*Geothlypis trichas*) and Hermit Thrush (*Catharus guttatus*) each have divergence times of less than 1 million years in North America but have *D* values above 0.7 (i.e., they are close to ignoring the other taxon). Such weak responses between related taxa at high latitudes contrast with strong ongoing responses between many pairs of allopatric Amazonian taxa considered full species and that often diverged from a common ancestor more than 2 million years ago (Fig 2).

Several features that may differ between the tropics and the temperate zone do not appear to contribute strongly to the slower loss of response in the tropics. First, vegetation may differ, generating different responses to songs of intruders. For example, if tropical vegetation is denser, males may need to come closer to the sound source to discover the individual. We worked in both forested (open and dense) and edge habitats in both regions (Methods) but can more directly reject this possibility because males in the temperate region and Amazon approached the speaker to a similar extent in response to contypic song (S1 Fig). Second, taxonomic make-up differs between the regions. For example, 80% of the species studied in Amazonia sing innate songs (mostly suboscines but some nonpasserines), whereas 80% of the species studied in North America have a learning component to their songs (oscines), and other studies have invoked learning as slowing the decay in response [29]. However, we found that including this variable in the models made little difference (Table 1). Finally, other features of the phenotype are used in species recognition [6,31]. Uy and Safran [31] conclude that

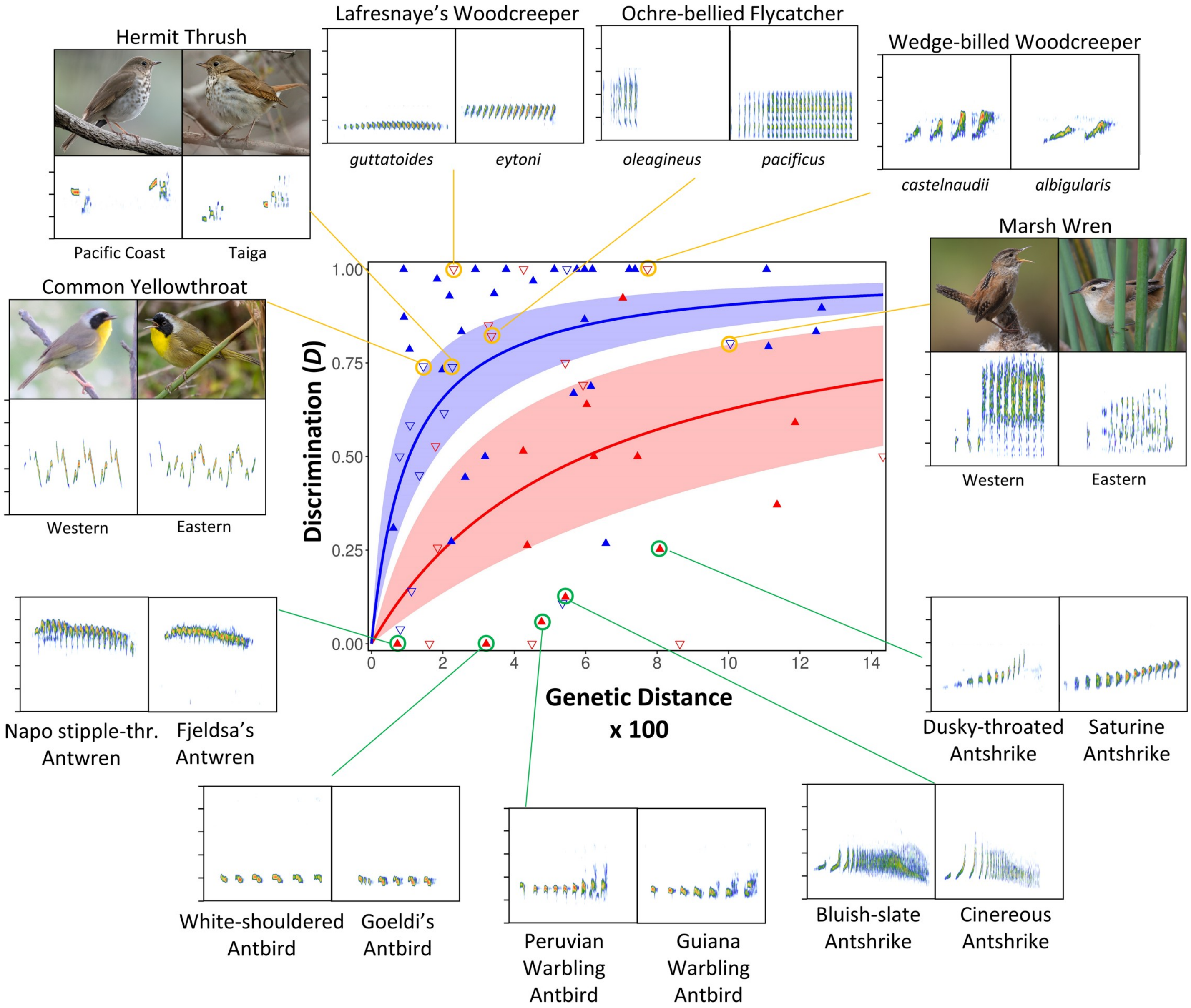

Fig 2. Song discrimination for Amazonian (red) and high latitude North American (blue) taxon pairs with at least two playback experiments performed. Excluding experiments with only a single playback experiment reduced the scatter but had limited impact on the best-fit relationship. The best-fit Michaelis–Menten curves and their 95% confidence bands are shown, calculated as in Fig 1A (statistics are in S2 Table). Intraspecific (open inverted triangles) and species-level (solid triangles) splits are shown separately. Yellow lines highlight selected intraspecific splits with discrimination scores exceeding 0.7. Green lines highlight selected allopatric or parapatric species-level splits with discrimination scores less than 0.3. Sonograms for highlighted taxon pairs plot song frequency (*y* axis, with each bar representing an increase of 2 kilohertz) versus time (*x* axis, with time shown at the same scale within a taxon pair but not between them), with warmer colors representing higher amplitude. Photos were taken from Flickr (www.flickr.com) under the CC-BY 2.0 license. Photo credits as follows. Common Yellowthroat: left, synspectrum; right, Andy Morffew. Hermit Thrush: left, Becky Matsubara; right, Matt McGillivray. Marsh Wren: left, Nigel; right, Andy Reago and Crissy McClarren. Playback data used to make this figure are in S1 Data. CC, Creative Commons.

https://doi.org/10.1371/journal.pbio.3000478.g002

plumage coloration is often a secondary short-range signal used in species recognition after vocalizations have provoked an initial response. In general, plumage and vocalizations are expected to act synergistically in species recognition, and in experiments, both contribute to responses [6,32]. Moreover, both traits diverge more slowly in the neotropics than at high

latitudes [3–5], so it is unlikely that either can bias the result away from slower rates of response loss in Amazonia.

Divergence in song cues

Reduced responses to heterotypic song at high latitudes may be a consequence of relatively large differences in songs compared to the tropics. Indeed, song frequency and song syllable diversity have both diverged at a slower rate in the neotropics than at higher latitudes [3,4] (S2 Fig), leading to the expectation that responses should be lost more slowly as well. In fact, responses only weakly correlate with differences between pairs in frequency and song length (Fig 1B and S3 Fig), and after accounting for frequency and song length differences, we found Amazonian species still exhibit significantly stronger song responses (Fig 1B and S3 Fig). Part of the explanation may be that other unmeasured aspects of song differ between species pairs, and these are less divergent in Amazonia. While we have not included all song variables, in other studies, frequency and song length appear to be features that birds are especially well-tuned to, with experimental modifications in either leading to a reduction in aggressive responses by males [33,34]. We conclude that responses may be lost more slowly in Amazonia because of slower song divergence there, but it is also likely that tropical species respond to a broader diversity of acoustic signals.

Year-round territoriality

Amazonia differs conspicuously from temperate North America in having a much less seasonal climate. Consequently, familiar conspecifics are in contact through much of the year, with two-thirds of the species we studied holding year-round territories (S1 Table). By contrast, at high latitudes, 93% of species in our dataset set up territories each year, often for a relatively short time (S1 Table). A short breeding season necessitates rapid mate choice and territory establishment mediated by song, especially in migratory species [35]. In contrast, species in tropical regions have little requirement for rapid recognition based on song cues and may regularly respond to and investigate somewhat different songs.

Beyond conspecifics, interspecific territoriality is associated with strong aggressive responses to playback of sympatric species in the Amazon, as well as in other aseasonal environments [28,36–38]. In Peru, Tobias and colleagues [26] found that during the dawn chorus, acoustically similar species sing in the same 10-minute window, suggesting advantages to song similarity, such as interspecific territoriality. In the Andes, species with year-round territorial defense form much sharper elevational replacements than nonterritorial or seasonally territorial species, attributed to year-round competitive (behavioral) interactions between species [39]. Interspecific territoriality is also present among sympatric species in temperate North America [40], but heterospecific song playbacks among sympatric species in temperate regions have not produced the kinds of strong aggressive responses recorded in the tropics [41]. Interspecific interactions in the tropics may thus lead to aggressive responses to novel songs from allopatric forms as well, consistent with our finding that year-round territoriality is especially strongly associated with a slow loss of response between allopatric forms (Table 1). Nevertheless, we found slower rates of loss of aggressive response in Amazonia are still apparent when species that do not hold year-round territories are compared (Table 1), regardless of whether rates are measured as a function of time (Table 1) or song divergence (S4 Table).

Consequences for speciation

Our results imply that slower song divergence and the presence of year-round territoriality contribute to slower loss of heterotypic response in Amazonia versus temperate North

America, although these two factors do not appear to explain the entire pattern. Regardless of the causes, a slower loss of response to song to related allopatric taxa in Amazonia may affect speciation rate in two key ways. These are first, through a slower establishment of secondary sympatry, and second, through a slower buildup of reproductive isolation in allopatry. We discuss each in turn.

A high aggressive response is likely to prevent establishment of foreign males [16,17] and thereby limit accumulation of closely related species in the same location [36,39]. Prolonged retention of aggression between related taxa may be one reason why the rate at which sister pairs have come into sympatry is lower in the neotropics than North America [13,14]. If sympatry is associated with reinforcement of premating reproductive isolation [19], a delay in establishment of sympatry will slow the completion of reproductive isolation. Further, because range expansions are essential to ongoing allopatric and parapatric modes of speciation [13,42,43], delayed attainment of sympatry should also contribute to slow speciation rates [13].

Assessments of latitudinal differences in speciation rate usually focus on development of reproductive isolation [44–47]. Such differences have been directly tested only once. In laboratory experiments with *Drosophila*, intrinsic postmating isolation has evolved more quickly in tropical species, perhaps because of the presence of endosymbionts [48]. We argue that, though our results do not directly test for this (we test male aggressive response rather than female mating response), they imply premating reproductive isolation in birds is likely to be evolving more slowly in allopatry in Amazonia than in temperate North America because female propensity to mate is likely to correlate with male responses. Direct experimental evidence for such a correlation is limited: female copulation solicitations to male song playbacks from different populations have been examined for just nine species [49]. In all cases, females give lower responses to songs from allopatric populations than songs from their own population. In eight of these studies, male aggressive responses are lower as well, implying that at this broad level, male aggression correlates with female attraction. In one case, females showed decreased copulation solicitation to the allopatric population's song, but males responded similarly (*Zonotrichia capensis* sparrows in the Andes [50]). This example suggests that females might sometimes discriminate against related allopatric forms even as territorial males give strong aggressive responses.

Among sympatric Amazonian species, many of which are long diverged from each other, interactions between interspecifically territorial species result in high levels of between-species aggression among males [28,36] but may also lead to females discriminating among very similar songs [28]. Such a dissociation of female discrimination from male heterospecific response likely results from a combination of avoidance of interacting species, reinforcement of premating isolation with related sympatric species, and other forms of reproductive interference. However, an ability of females to discriminate songs of sympatric heterospecific males in Amazonia does not necessarily imply they will fail to mate with males from closely related allopatric taxa, which are expected to be similar in many traits to those of males from their own population (including song, color, and call notes). Because these songs are rarely experienced, these similarities may result in female investigation, response, and hybridization, corresponding with high levels of male aggression to such songs, as mimicked by our experiments.

Assays of female copulation responses are probably the best way to more directly ask if females are losing propensity to mate across taxa more slowly in Amazonia than in temperate regions. However, two other lines of evidence suggest that premating reproductive isolation is evolving relatively slowly in the neotropics. First, exceptionally old taxa form hybrid zones in the neotropics [13,51,52], demonstrating that females fail to consistently discriminate heterospecifics from conspecifics. Across one suture zone in a headwater region of the Amazon, detailed field sampling demonstrated parapatric contact between seven species pairs ranging

in age up to about 4 million years of separation. All species pairs formed hybrids along these parapatric contact zones [51]. These results imply that females in Amazonia have evolved limited premating isolation with their close allopatric relatives. In the oldest of these parapatric pairs, genomic data are consistent with random mating after 4 million years [52]. Hybrid zones are narrow (10 to 25 km), and concordant geographic clines across hundreds of loci imply the presence of strong intrinsic postmating isolation [52], generally expected to be present given these long divergence times [53]. In contrast, hybrid zones are rare between old taxon pairs above 45 degrees latitude [13].

A second line of evidence pointing to slow evolution of premating isolation in allopatry in Amazonia is that many traits used as premating cues, including songs [this study, 3, 4], body size [2], and plumage color [5], are evolving more slowly toward the equator. A currently low rate of evolution of premating isolation in Amazonia appears to be paradoxical given its high species richness, especially because many attempts to explain high tropical richness have been couched in terms of potentially high rates of acquisition of reproductive isolation [44,45,54,55], but relatively low current speciation rates in the tropics have been observed across diverse taxa [1,46,47]. Our results suggest the possibility that as species accumulate in sympatry, a wider window of response, coupled with a low rate of divergence in cues used for premating isolation [2], contributes to a slowing in the achievement of range overlaps between incipient species, in the development of premating isolation, and ultimately, in speciation rates in the neotropics.

Methods

Field protocols

Field-based song playback experiments targeted birds during breeding times. Unlike high latitudes, breeding in much of Amazonia is not strongly seasonal. Many species breed all year or for many months of the year, with October to December representing a period of somewhat increased breeding propensity in central Amazonia [56]. Acyclic breeding was the norm at a location near our Peruvian field sites [57].

JTW performed song playbacks in the lowlands of Amazonia (Loreto, Peru: 2009; Para, Brazil: 2012; Mato Grosso, Brazil: 2018) and in temperate and boreal regions of North America (British Columbia, Canada: 2010; Ontario, Canada: 2010, 2011; Quebec, Canada: 2011; Indiana, California, Oregon, and Washington, USA: 2010) on 109 pairs of taxa (sister species identified from molecular phylogenies, pairs of genetically differentiated phylogroups, or pairs of closely related congeners that belong to a superspecies complex) representing 81 species complexes (S1 Data). He attempted to test all such pairs for which we could obtain song and genetic data and that he encountered in the field. Phylogroup taxa were included so as not to bias analyses by ignoring taxa pairs that may not evolve as rapidly in traits like song that may be used by taxonomists in species-level classification. Most taxon pairs were allopatric (non-overlapping geographically, which in the case of the Amazon often meant separated by the Amazon, Napo, or other rivers) or parapatric (meeting along narrow contact zones no wider than 100 km), but 20 sympatric sister species were also tested (overlapping by more than 100 km). In analyses in which we estimate response during allopatric phases of speciation, we included allopatric and parapatric taxa and excluded the 20 sympatric ones.

JTW worked in North America during the breeding season from April to July, primarily in coniferous or mixed coniferous and deciduous forests in northern California and Oregon; in coastal and interior British Columbia, Ontario, and Quebec; and in deciduous forests of Indiana. Experiments were also performed at forest edge and a small number in adjacent grasslands or marshlands. Experiments in Amazonia were conducted mostly in September and

October in lowland wet forests (varzea, white-sands forest, and terra firme) and their edge habitats in the Loreto Department of Peru at a series of sites on opposite sides of the Amazon and Napo Rivers, and a small number of experiments were conducted in terra firme and seasonal forests and their edge habitats of the states of Para and Mato Grosso, Brazil during the months of February to May. In each location, JTW searched opportunistically for males (usually singing) from shortly after dawn until shortly before dusk and, once males were located, initiated the experimental protocol.

Playback experiments used recordings of song (including mechanical display noises produced by hummingbird tails during displays and territorial drumming of woodpeckers) obtained from our own field recordings, the Macaulay Library, xeno-canto.org, commercial recordings, and private collections. Playback files were generated prior to field work for a large pool of potential avian pairs of taxa. Song files were edited in RAVEN PRO (website: www.birds.cornell.edu/raven) to exclude or minimize background noises, shorten long breaks between song bouts, standardize song amplitude, and eliminate calls interspersed with song. The social context of many call types renders them unsuited for testing species responses because they often serve as alarms and are used to attract heterospecifics when mobbing predators [58]. For these reasons, we felt it imperative to eliminate calls from recordings in order to isolate the effect of song on species responses.

Each playback experiment lasted a total of 260 seconds and included 120 seconds of playback of an experimental song (i.e., the song of the heterotypic taxon), followed by a 10-second pause and then 120 seconds of playback of the control song (a song from a contypic population that, when possible, was obtained from a geographic region close to the focal bird but never represented a recording of the focal bird itself), followed by another 10 seconds during which the response continued to be recorded. We used only a 10-second pause between experimental and control playbacks. Others have used a several-minute pause, but we found that doing so resulted in many birds leaving the vicinity of playback with the risk that they would not respond during playback of the control. Importantly, playback of the target species vocalizations was not performed prior to the initiation of playback experiments because doing so might excite the bird such that it would then respond to subsequent playback of the experimental song even if it would not normally do so. We always performed playback of the experimental song before the control song for the same reasons. Note that by consistently playing the control after the experimental song, we may be affecting overall response to the control (for example, if the bird responds aggressively to the experimental song, it may continue to respond aggressively to the control). However, we are here concerned with comparisons between Amazonia and temperate regions, and our protocols were identical across latitudes.

When sufficient numbers of song files were available, we made more than one set of playback experiments from different song files. Playback experiments were conducted by setting a Pignose 7–100 portable speaker (Pignose, Las Vegas, NV, USA) generally 20 m to 30 m from the target bird, with the observer located about 10 m from the speaker. The closest approach to the speaker (measured only while a bird is perched) and the number of flyovers and flybys of the speaker were recorded for 120 seconds of playback of the experimental song and for the following 10-second pause, and the same was done for the control song and its subsequent 10-second pause. JTW has a 1-m stride. Approach distances less than 10 m were generally measured by walking the distance and counting strides, though vegetation did not always allow this, and some distances were estimated. Longer distances were generally estimated. A flyover involves the bird flying directly over the speaker (0 to 1.5 meters horizontal distance from the speaker), while a flyby involves the bird flying directly past the speaker region but not directly over it (typically 1.5 m to 4 m horizontal distance from the speaker). Only a single playback experiment was performed in each territory to ensure that replicate experiments on

the same species were statistically independent. Effectively, this meant spacing playback experiments every 100 m to 200 m and not repeating experiments in those localities.

Genetic divergence estimates

Relative evolutionary ages of pairs of taxa were approximated using GTR gamma distances obtained for at least one of three mitochondrial genes: cytochrome b (Cyt b), NADH dehydrogenase 2 (ND2), and cytochrome oxidase I (COI). Cyt b is reported to evolve at a rate of approximately 2% per million years [30]. We performed least-squares regression fit through the origin of Cyt b versus ND2 or COI. Genetic distances of ND2 and COI were divided by the slope of these fits (slope = 1.1 for ND2 and 0.97 for COI) so that they were comparable to distances for Cyt B, and then the average genetic distance across these genes was obtained. Some estimates may be affected by introgression across the species boundaries, but we have no reason to suspect this is more likely in temperate regions than in the tropics.

Song divergence estimates

We calculated song divergence for each pair of taxa based on the song files used in playback experiments but excluded songs comprised of mechanical sounds (for example, woodpecker drumming).

We used the *dfreq* function of the R package seewave [59] to extract the dominant frequency at each time point for every spectrogram trace (for example, roughly equivalent to a note) for each song within a song file. Song length (measured between the 10th and 90th percentile of energy), length ratio (the proportional time of a song in which sound was produced), and 10 measurements of song frequency were obtained as follows: low frequency (measured at the 5th percentile of energy), high frequency (measured at the 95% percentile of energy), median frequency, 90th percentile bandwidth (measured between the 5th and 95th percentile frequencies) and 50th percentile bandwidth (measured between the first and third quartile frequencies), frequency variance, three measures of frequency autocorrelation (measured after at a repeating interval of 0.1, 0.05, and 0.01 seconds), and the autocorrelation proportional time (the proportional time point in the song at which autocorrelation first reaches 0.5, which is set to 1 for songs in which autocorrelation never reaches 0.5). Autocorrelation was measured using the R function *acf* [60] and was obtained with the soundless spaces between spectrogram traces removed. Autocorrelation helps determine how rapidly frequency changes through time in a song. High values occur when frequency changes little through the course of a song.

Measures were averaged across songs within a song file, and principal component analyses (PCAs) were performed on log-transformed data (all measures were log-transformed except autocorrelation metrics). Two separate PCAs were performed—the first on all measures (principal component [PC] 1 to PC3 accounted for 41%, 21%, and 15% of the variance, respectively; see S4A Fig for loadings), the second on only frequency measures (PC1 to PC3 accounted for 49%, 24%, and 10% of the variance; see S4B Fig for loadings). The latter PCA was performed in order to isolate the effect of frequency from song length because frequency measures were loaded on multiple PCs in the first PCA. Each PC score was standardized by dividing its values by the relevant standard deviation. Values were then averaged across different song files for each taxon, and Euclidean distances of the first three standardized PCs were obtained for each of the two PCAs. A third Euclidean distance was obtained for log-transformed song length. These three measures were used as metrics of song divergence.

Quantification and statistical analysis

We quantified two responses during playback experiments: the closest approach to the speaker (measured as horizontal distance, not vertical) and the number of flying passes made over or near the speaker. Both responses were quantified as the response to experimental playback relative to the response to control playback. An approach response was calculated only for experiments in which males came within 10 m of the speaker during playback of the experimental song, control song, or both. For such experiments, approach to the speaker is measured as

$$M_1 = \max[0, 1 - A_e/A_c], \quad (1)$$

where A_e is an "approach score" to experimental playback and A_c is the approach score to control playback. Approach scores are calculated as max(0, 1 − C/10), where C is the closest perched approach in meters. Approach scores range from 0 to 1, with a larger value indicating a stronger response. A value of 0 for the approach score indicates the bird did not approach closer than 10 m from the speaker, which we consider to be no response, while a value of 1 indicates the male perched on or vertically above the speaker (i.e., a strong response). A value of 1.0 for M_1 occurs when the male failed to come closer than 10 m in response to heterotypic song while it came within 10 m in response to contypic song (i.e., no response to heterotypic song), and a value of 0 occurs when the bird approached to an equal distance less than 10 m during experimental and control playback. Values between 0 and 1 indicate the male approached to less than 10 m during both heterotypic and contypic song, but the approach was closer for the contypic. M_1 equals 0 for experiments when the bird comes closer for the experimental song than the control song. If the position of the bird was less than 10 m at the start of the experiment, M_1 was calculated only if the bird moved even closer to the speaker during either experimental or control song (all such cases). We did not calculate M_1 for seven experiments in which the bird failed to approach less than 10 m for either experimental or control playback and for two experiments in which the closest approach was not observed.

Fly-pasts are measured as

$$M_2 = \max[0, 1 - F_e/F_c], \quad (2)$$

where F_e and F_c are a measure of the number of flights past the speaker during the experimental and control playback, respectively, and are calculated as the number of flybys plus twice the number of flyovers. Passes directly over the speaker represent a stronger response than vicinity passes and are thus given double the weight. M_2 is given a value of 0 when F_c is equal to 0 (for example, when flying passes occur only for the experimental playback) and is only calculated when at least one fly-pass occurred during either the experimental or control playback. M_2 varies from 0 to 1. A value of 0 indicates equal response to heterospecific and control playback, and a value of 1 indicates the response only to the control.

We calculated a measure of total response (D) as the average value of M_1 and M_2 for an experiment. For experiments lacking a response for one of these measures (i.e., not all approaching males respond with flybys, and not all males approach within 10 m even when performing flybys), the value of the other measure was used as the response score. Experiments lacking a response for both measures were not recorded. Both measures were averaged in 184 experiments, while M_1 only was used in nine experiments and M_2 only in 52 experiments. D ranges from 0 to 1. A value of 0 represents a strong response to the heterotypic form, which occurs when a bird responds at least as strongly to experimental playback as it does to control playback. A value of 1 implies the individual failed to respond to experimental playback but did respond to control playback. D was averaged across replicates of the same experiment (involving the same experimental and control song files) and then across statistically distinct

experiments involving different experimental and control song files. For 19 pairs of taxa, playback experiments were performed on each of the two taxa. Because responses for the two taxa were significantly correlated (Pearson's $r = 0.61$, $p = 0.0057$), we averaged them to obtain a single response for the pair.

We quantified the accumulation of D as a function of time using a modeling framework based on the Michaelis–Menten curve borrowed from enzyme kinetics as follows:

$$D = T/(\beta + T), \quad (3)$$

where T is genetic distance and β represents the value of T when D is 0.5. D at $T = 0$ will be 0, and the curve will asymptote at $D = 1$. While other asymptotic models could have been used, we chose this model because it has previously been applied to estimating rates of response loss [29] and because the β statistic provides an intuitive measure of the rate at which response to heterospecific song is lost. β measures the time required for response (D) to reach a value of 0.5. A larger value of β indicates a slower rate of song discrimination. Eq 3, measured across all taxon pairs, can be compared to a model in which tropical and temperate pairs each receive separate curves (for example, distinct β-values), taxon pairs with other features of interest (i.e., innate or learned song or presence or absence of year-round territoriality) each received separate curves, or a joint model in which these features each received separate curves both at high latitudes and in Amazonia.

Fourteen of the 81 species complexes tested had two or more pairs of taxa sampled within them, and these are not statistically independent because playbacks from multiple taxa were performed to the same taxon, resulting in some pairs of taxa being phylogenetically nested within other pairs (S5 Fig). All statistical analyses were designed to account for this lack of independence by generating 1,000 datasets, each with only a single taxon pair drawn at random from each species complex. We fitted the above four models to each of these 1,000 datasets and used a bootstrap of each to estimate 95% confidence intervals for β. Bootstraps were drawn in such a way as to retain the number of sister pairs in each discrete category used by a given model (for example, Amazonia versus high latitude, learned versus innate song, or the combined model of latitude and song transmission mode).

We also quantified the accumulation of D as a function of song divergence using the Michaelis–Menten curve in which a parameter z is added that shifts the curve along the axis of song divergence, thereby accommodating a nonzero intercept while still allowing song discrimination to increase asymptotically. This parameter is required when song divergence is the predictor because each metric of song divergence is unable to capture all aspects of song that may differ, and thus, there is no expectation that $D = 0$ when song divergence for a given metric equals 0. The Michaelis–Menten curve with the z parameter is given by

$$D = (S - z)/(\beta + S - 2z), \quad (4)$$

where S is song divergence and β is the value of S when D is 0.5.

Michaelis–Menten curves were fitted using nonlinear least squares using the *nls* R function. Finally, we fitted a Brownian motion model to measure the evolutionary rate (δ^2) of song divergence as a function of genetic distance using the R package EvoRAG [61].

Supporting information

S1 Fig. Histograms of responses to playback, all experiments combined. Left: the number of flyovers and flybys across the speaker during playback of (A) heterotypic and (B) contypic songs and (C) the ratio between A and B used to quantify relative level of heterotypic discrimination. Ratios greater than 1 are set to 1 indicating a lack of discrimination, while values of 0

indicate response only to conspecific playback. Right: the closest approach to the speaker during playback of (D) heterotypic and (E) contypic song and (F) the ratio of conspecific to heterospecific approach as an indicator of heterospecific discrimination used to quantify relative level of heterotypic discrimination. Ratios of 0 indicate approach only to conspecific. Ratios of 1 indicate equal response to conspecific and heterospecific. Data used to make this figure are in S1 Data.
(TIF)

S2 Fig. Rates of song evolution in tropical and temperate birds. Song evolves faster at higher latitudes for (A) overall song divergence as measured by Euclidean distances of PC1 to PC3 for 12 measures of song timing and frequency and (B) song frequency as measured by Euclidean distances for PC1 to PC3 for 10 measures of frequency but not (C) Euclidean distances of log-transformed song length. Lines show the mean evolutionary rate (δ^2) under a Brownian motion model obtained from 1,000 datasets that randomly sampled with replacement one sister pair per species complex. Shading show 95% confidence bands obtained from 1,000 bootstrap replicates. Data used to make this figure are in S1 Data. PC, principal component.
(TIF)

S3 Fig. Discrimination as a function of song divergence. Song divergence is measured using a Euclidean distance of PC1 to PC3 obtained from 10 measures of frequency and its autocorrelation (A), and song length is measured using the Euclidean distance of log-transformed song lengths (B). Lines and shading as in Fig 1. Though confidence bands overlap across bootstraps, discrimination was greater in the temperate than Amazonia in 100% of bootstrap replicates for (A), 97.1% for (B), 100% for (C), and 95% for (D). Data used to make this figure are in S1 Data. PC, principal component.
(TIF)

S4 Fig. PC loadings of song measures. Loadings for PCA of (A) all 12 measures of song frequency and length and (B) 10 measures of frequency and its autocorrelation. Eigenvalues and percent variance explained by each PC are indicated above each panel. PC, principal component; PCA, principal component analysis.
(TIF)

S5 Fig. Example of phylogenetically nested taxon pairs tested within the *Oreothlypis ruficapilla* species complex. Arrows start at the taxon on which playback was performed and point to the taxon whose song was tested. Arrow colors correspond to their corresponding colored nodes on the phylogeny. For the Nashville Warbler and Calaveras Warbler species pair, playbacks were performed on both species. In all such cases, the average response was used as the metric of discrimination for the species pair. Nested species pairs are not statistically independent. Thus, for each analysis in this paper, 1,000 datasets were generated, each with a single taxon pair drawn at random from each species complex. Statistics were then performed on these datasets. For bootstrap analysis, a single bootstrapped dataset was generated from each randomized dataset.
(TIF)

S1 Table. Social systems for species in our dataset.
(DOCX)

S2 Table. Support for models of song discrimination evolution as a function of genetic distance using the Michaelis–Menten modeling framework using only sister pairs with two or more playback experiments.
(DOCX)

S3 Table. Support for models of song evolution as a function of genetic distance using Brownian motion models.
(DOCX)

S4 Table. Support for models of song discrimination evolution as a function of song divergence.
(DOCX)

S1 Data. The dataset used in this paper is provided as a supporting online file, Dataset 1.
(XLSX)

S1 Code. R code of models tested using the Michaelis–Menten framework.
(TXT)

S2 Code. Example text file used to illustrate the code.
(TXT)

Acknowledgments

Field work was conducted at four field stations operated by Project Amazonas (the owners and staff of which also aided greatly with field logistics) in Peru and on property made available by a number of private landowners in Brazil. Omar Yossofzai helped tabulate field notes. Mauricio Ugarte Lewis, Alexandre Alexio, Laura Weir, James Weir, Joanne Weir, Susie Ha, and Ken Ha assisted with field logistics.

Author Contributions

Conceptualization: Jason T. Weir, Trevor D. Price.

Data curation: Jason T. Weir.

Formal analysis: Jason T. Weir.

Funding acquisition: Jason T. Weir, Trevor D. Price.

Investigation: Jason T. Weir.

Methodology: Jason T. Weir.

Project administration: Jason T. Weir.

Validation: Jason T. Weir.

Visualization: Jason T. Weir, Trevor D. Price.

Writing – original draft: Jason T. Weir, Trevor D. Price.

Writing – review & editing: Jason T. Weir, Trevor D. Price.

References

1. Weir J, Schluter D. The latitudinal gradient in recent speciation and extinction rates of birds and mammals. Science. 2007; 315: 1574–1576. https://doi.org/10.1126/science.1135590 PMID: 17363673
2. Lawson A, Weir J. Latitudinal gradients in climatic-niche evolution accelerate trait evolution at high latitudes. Ecol Lett. 2014; 17: 1427–1436. https://doi.org/10.1111/ele.12346 PMID: 25168260
3. Weir J, Wheatcroft D, Price T. The role of ecological constraint in driving the evolution of avian song frequency across a latitudinal gradient. Evolution. 2012; 66: 2773–2783. https://doi.org/10.1111/j.1558-5646.2012.01635.x PMID: 22946802

4. Weir J, Wheatcroft D. A latitudinal gradient in rates of evolution of avian syllable diversity and song length. Proc R Soc B-Biol Sci. 2011; 278: 1713–1720.
5. Martin P, Montgomerie R, Lougheed S. Rapid sympatry explains greater color pattern divergence in high latitude birds. Evolution. 2010; 64: 336–347. https://doi.org/10.1111/j.1558-5646.2009.00831.x PMID: 19744123
6. Price T. Speciation in Birds. Boulder, CO.: Roberts and Co.; 2008.
7. Coyne J, Orr H. Speciation. Sunderland, Mass.: Sinauer; 2004.
8. Parker T, Greig E, Nakagawa S, Parra M, Dalisio A. Subspecies status and methods explain strength of response to local versus foreign song by oscine birds in meta-analysis. Anim Behav. 2018; 142: 1–17.
9. Irwin D, Bensch S, Price T. Speciation in a ring. Nature. 2001; 409: 333–337. https://doi.org/10.1038/35053059 PMID: 11201740
10. Alström P, Ranft R. The use of sounds in avian systematics and the importance of bird sound archives. Bull Br Ornithol Club Suppl. 2003; 123A: 114–135.
11. Uy J, Moyle R, Filardi C. Plumage and song differences mediate species recognition between incipient flycatcher species of the Solomon Islands. Evolution. 2009; 63: 153–164. https://doi.org/10.1111/j.1558-5646.2008.00530.x PMID: 18803681
12. Freeman B, Montgomery G. Using song playback experiments to measure species recognition between geographically isolated populations: A comparison with acoustic trait analyses. The Auk. 2017; 134: 857–870.
13. Weir J, Price T. Limits to speciation inferred from times to secondary sympatry and ages of hybridizing species along a latitudinal gradient. Am Nat. 2011; 177: 462–469. https://doi.org/10.1086/658910 PMID: 21460568
14. Pigot A, Tobias J, Jetz W. Energetic constraints on species coexistence in birds. PLoS Biol. 2016; 14(3): e1002407. https://doi.org/10.1371/journal.pbio.1002407 PMID: 26974194
15. Grether G, Anderson C, Drury J, Kirschel A, Losin N, Okamoto K, et al. The evolutionary consequences of interspecific aggression: Aggression between species. Ann N Y Acad Sci. 2013; 1289: 48–68. https://doi.org/10.1111/nyas.12082 PMID: 23601031
16. Pearson S, Rohwer S. Asymmetries in male aggression across an avian hybrid zone. Behav Ecol. 2000; 11: 93–101.
17. Scordato E. Geographical variation in male territory defence strategies in an avian ring species. Anim Behav. 2017; 126: 153–162.
18. Servedio M, Noor M. The role of reinforcement in speciation: theory and data. Annu Rev Ecol Evol Syst. 2003; 34: 339–364.
19. Hudson E, Price T. Pervasive reinforcement and the role of sexual selection in biological speciation. J Hered. 2014; 105: 821–833. https://doi.org/10.1093/jhered/esu041 PMID: 25149257
20. Gröning J, Hochkirch A. Reproductive interference between animal species. Q Rev Biol. 2008; 83: 257–282. PMID: 18792662
21. Grether G, Peiman K, Tobias J, Robinson B. Causes and consequences of behavioral interference between species. Trends Ecol Evol. 2017; 32: 760–772. https://doi.org/10.1016/j.tree.2017.07.004 PMID: 28797610
22. Tobias J, Sheard C, Seddon N, Meade A, Cotton A, Nakagawa S. Territoriality, social bonds, and the evolution of communal signaling in birds. Front Ecol Evol. 2016; 4: 74.
23. Terborgh J, Robinson S, Parker T, Munn C, Pierpoint N. Structure and organization of an Amazonian forest bird community. Ecol Monogr. 1990; 60: 213–238.
24. Tobias J, Cornwallis C, Derryberry E, Claramunt S, Brumfield R, Seddon N. Species coexistence and the dynamics of phenotypic evolution in adaptive radiation. Nature. 2014; 506: 359–363. https://doi.org/10.1038/nature12874 PMID: 24362572
25. Tobias J, Seddon N. Signal design and perception in Hypocnemis antbirds: evidence for convergent evolution via social selection. Evolution. 2009; 63: 3168–3189. https://doi.org/10.1111/j.1558-5646.2009.00795.x PMID: 19659594
26. Tobias J, Planque R, Cram D, Seddon N. Species interactions and the structure of complex communication networks. Proc Natl Acad Sci USA. 2014; 111: 1020–1025. https://doi.org/10.1073/pnas.1314337111 PMID: 24395769
27. Kirschel A, Blumstein D, Cohen R, Buermann W, Smith T, Slabbekoorn H. Birdsong tuned to the environment: green hylia song varies with elevation, tree cover, and noise. Behav Ecol. 2009; 20: 1089–1095.
28. Seddon N, Tobias J. Character displacement from the receiver's perspective: species and mate recognition despite convergent signals in suboscine birds. Proc R Soc B-Biol Sci. 2010; 277: 2475–2483.

29. Freeman B, Montgomery G, Schluter D. Evolution and plasticity: Divergence of song discrimination is faster in birds with innate song than in song learners in Neotropical passerine birds. Evolution. 2017; 71: 2230–2242. https://doi.org/10.1111/evo.13311 PMID: 28722748

30. Weir J, Schluter D. Calibrating the avian molecular clock. Mol Ecol. 2008; 17: 2321–2328. https://doi.org/10.1111/j.1365-294X.2008.03742.x PMID: 18422932

31. Uy J, Safran R. Variation in the temporal and spatial use of signals and its implications for multimodal communication. Behav Ecol Sociobiol. 2013; 67: 1499–1511.

32. Baker M, Baker A. Reproductive behavior of female buntings—isolating mechanisms in a hybridizing pair of species. Evolution. 1990; 44: 332–338. https://doi.org/10.1111/j.1558-5646.1990.tb05202.x PMID: 28564380

33. Nelson D. Feature weighting in species song recognition by the field sparrow (Spizella pusilla). Behaviour. 1988; 106: 158–182.

34. Linhart P, Slabbekoorn H, Fuchs R. The communicative significance of song frequency and song length in territorial chiffchaffs. Behav Ecol. 2012; 23: 1338–1347.

35. Catchpole C. Sexual selection and the evolution of complex songs among European warblers of the genus Acrocephalus. Behaviour. 1980; 74: 149–166.

36. Robinson S, Terborgh J. Interspecific aggression and habitat selection by Amazonian birds. J Anim Ecol. 1995; 64: 1–11.

37. Jankowski J, Robinson S, Levey D. Squeezed at the top: Interspecific aggression may constrain elevational ranges in tropical birds. Ecology. 2010; 91: 1877–1884. https://doi.org/10.1890/09-2063.1 PMID: 20715605

38. Freeman BG Freeman A, Hochachka W. Asymmetric interspecific aggression in New Guinean songbirds that replace one another along an elevational gradient. Ibis. 2016; 158: 726–737.

39. Freeman B, Tobias J, Schluter D. Behavior influences range limits and patterns of coexistence across an elevational gradient in tropical bird diversity. Ecography. 2019. https://doi.org/10.1111/ecog.04606 Available from: https://onlinelibrary.wiley.com/doi/abs/10.1111/ecog.04606.

40. Losin N, Drury J, Peiman K, Storch C, Grether G. The ecological and evolutionary stability of interspecific territoriaity. Norris R, editor. Ecol Lett. 2016; 19: 260–267. https://doi.org/10.1111/ele.12561 PMID: 26757047

41. Noon B. The distribution of an avian guild along a temperate elevational gradient: the importance and expression of competition. Ecol Monogr. 1981; 51: 105–124.

42. Grant P, Grant B. Speciation and hybridization in island birds. Philos Trans R Soc Lond Ser B–Biological Sci. 1996; 351: 765–772.

43. Price T, Hooper D, Buchanan C, Johansson U, Tietze D, Alstrom P, et al. Niche filling slows the diversification of Himalayan songbirds. Nature. 2014; 509: 222–225. https://doi.org/10.1038/nature13272 PMID: 24776798

44. Allen A, Gillooly F. Assessing latitudinal gradients in speciation rates and biodiversity at the global scale. Ecol Lett. 2006; 9: 947–954. https://doi.org/10.1111/j.1461-0248.2006.00946.x PMID: 16913938

45. Schemske D. Biotic interactions and speciation in the tropics. In: Butlin RK, Bridle JR, Schluter D, editors. Speciation and Patterns of Diversity. Cambridge, United Kingdom: Cambridge University Press; 2009. pp. 219–239.

46. Schluter D. Speciation, ecological opportunity, and latitude. Am Nat. 2016; 187: 1–18. https://doi.org/10.1086/684193 PMID: 26814593

47. Rabosky D, Chang J, Title P, Cowman P, Sallan L, Friedman M, et al. An inverse latitudinal gradient in speciation rate for marine fishes. Nature. 2018; 559: 392–395. https://doi.org/10.1038/s41586-018-0273-1 PMID: 29973726

48. Yukilevich R. Tropics accelerate the evolution of hybrid male sterility in Drosophila. Evolution. 2013; 67: 1805–1814. https://doi.org/10.1111/evo.12056 PMID: 23730771

49. Uy J, Irwin D, Webster M. Behavioral isolation and incipient speciation in birds. Annu Rev Ecol Evol Syst. 2018; 49: 1–24.

50. Danner J, Danner R, Bonier F, Martin P, Small T, Moore I. Female, but not male, tropical sparrows respond more strongly to the local song dialect: implications for population divergence. Am Nat. 2011; 178: 53–63. https://doi.org/10.1086/660283 PMID: 21670577

51. Weir J, Faccio M, Pulido-Santacruz P, Barrera-Guzma A, Aleixo A. Hybridization in headwater regions, and the role of rivers as drivers of speciation in Amazonian birds. Evolution. 2015; 69: 1823–1834. https://doi.org/10.1111/evo.12696 PMID: 26095719

52. Pulido-Santacruz P, Aleixo A, Weir J. Morphologically cryptic Amazonian bird species pairs exhibit strong postzygotic reproductive isolation. Proc R Soc B-Biol Sci. 2018; 285: 20172081.

53. Price T, Bouvier M. The evolution of F1 postzygotic incompatibilities in birds. Evolution. 2002; 56: 2083–2089. PMID: 12449494

54. Mittelbach G, Schemske D, Cornell H, Allen A, Brown J, Bush M, et al. Evolution and the latitudinal diversity gradient: speciation, extinction and biogeography. Ecol Lett. 2007; 10: 315–331. https://doi.org/10.1111/j.1461-0248.2007.01020.x PMID: 17355570

55. Salisbury C, Seddon N, Cooney C, Tobias J. The latitudinal gradient in dispersal constraints: ecological specialisation drives diversification in tropical birds. Ecol Lett. 2012; 15: 847–855. https://doi.org/10.1111/j.1461-0248.2012.01806.x PMID: 22639858

56. Stouffer P, Johnson E, Bierregaard R, al et. Breeding seasonality in central Amazonian rainforest birds. The Auk. 2013; 130: 529–540.

57. Tallman D, Tallman E. Timing of breeding by antbirds (Formicadiidae) in an aseasonal environment in Amazonian Ecuador. In: Remsen JJV, editor. Studies in Neotropical Ornithology Honoring Ted Parker Ornithological Monograph, no 48. 1997. pp. 783–789.

58. Wheatcroft D, Price T. Rates of signal evolution are associated with the nature of interspecific communication. Behav Ecol. 2015; 26: 83–90.

59. Sueur J, Aubin T, Simonis C. Seewave: a free modular tool for sound analysis and synthesis. Bioacoustics. 2008; 18: 213–226.

60. RC Team. R: A language and environment for statistical computing. Vienna, Austria; 2014. Available from: http://www.r-project.org/.

61. Weir J, Lawson A. Evolutionary rates across gradients. Methods Ecol Evol. 2015; 6: 1278–1286.
